# Treating Alcohol Problems in the Context of Other Drug Abuse

**Published:** 1996

**Authors:** William R. Miller, Melanie E. Bennett

**Affiliations:** William R. Miller, Ph.D., is Regents’ Professor of Psychology and Psychiatry and Melanie E. Bennett, Ph.D., is a visiting assistant professor in the Department of Psychology and the Center on Alcoholism, Substance Abuse, and Addictions (CASAA), University of New Mexico, Albuquerque, New Mexico

**Keywords:** AOD dependence, multiple drug use, illicit drug, AOD associated consequences, causes of AODU (alcohol and other drug use), patient assessment, motivation, treatment factors, treatment method, treatment outcome, AODD (alcohol and other drug use disorder) relapse

## Abstract

People seeking treatment for alcohol problems frequently abuse other drugs as well, such as tobacco, cocaine, marijuana, methamphetamine, and opiates. The problem of polydrug use raises important issues for treatment providers. A person who abuses multiple drugs may have a more difficult time stopping drinking and a higher risk for relapse to alcohol use after treatment. Conversely, a person who successfully stops drinking may offset this achievement by substituting another drug for alcohol. Successful treatment must take into account both alcohol- and drug-related issues, with particular emphasis on assessment, motivation, treatment design, and outcome evaluation.

People who meet alcohol abuse or dependence criteria^1^ often use, abuse, or are dependent on other drugs as well. The “pure alcoholic” represents the minority in clinical settings, where the majority of patients engage in polydrug use. According to one recent study, more than 60 percent of men and women presenting for treatment of alcohol problems used another drug at least weekly (Caetano and Weisner 1995), and other studies have reported similarly high rates of multiple drug use (Weisner 1992*a*). Even in the general population, people who are alcohol dependent have been found to be five times more likely than other people (18 versus 3.5 percent) to meet the criteria for another drug-use disorder (Helzer and Pryzbeck 1988).

People with alcohol problems are more likely to use a variety of other drugs, including tobacco, cocaine, marijuana, methamphetamine, and opiates (for further details, see box, p. 120). Compared with individuals who experience only alcohol problems, polydrug users are likely to be younger and to have more severe alcohol and other drug (AOD)-related problems (Caetano and Weisner 1995; Hesselbrock et al. 1985). In an attempt to understand the high rate of comorbidity, some researchers have suggested that alcohol-use disorders and other drug-use disorders may share a common genetic (Jang et al. 1995) or psychological basis (Miller and Brown 1991) or that people using alcohol-drug combinations are attempting to self-medicate a psychological disorder (Jensen et al. 1990).

**Drugs of Abuse**

Drugs of abuse—whether they are banned, controlled (e.g., prescribed by physicians), or legal—produce a “high” or altered state of consciousness in people, and all are at least psychologically, if not physiologically, addictive. Here the similarities end, however. Different drugs produce a wide range of effects, and each can be categorized based on its overall effect (e.g., whether it is a stimulant or depressant). Following are descriptions of some commonly abused drugs classified according to the effects they produce:

- *Depressants—*Alcohol, sleeping medications (e.g., barbiturates), benzodiazepines (e.g., Valium^®^), and antianxiety drugs (i.e., minor tranquilizers) produce depressant or anesthetic effects by dampening the activity of brain tissue responsible for excitation, or stimulation. They also are capable of reducing pain (i.e., they have slight analgesic properties).
- *Stimulants—*Cocaine, amphetamines, and many weight-loss drugs (e.g., methylphenidate, or Ritalin^®^) produce stimulant effects through a variety of mechanisms in the brain. Caffeine and the nicotine in tobacco are less potent stimulants.
- *Narcotic analgesics—*Opiates, such as opium, morphine, codeine, and heroin, produce pain-killing effects in the body. Opiates are medically important because of this property but are subject to abuse as a result of it. Narcotic analgesics also produce drowsiness, cause mood changes, and, at high doses, affect mental functioning.
- *Hallucinogens and Marijuana—*Marijuana (i.e., cannabis); LSD (lysergic acid diethylamide); natural hallucinogens, such as mescaline and peyote; and related drugs, such as PCP (phencyclidine), produce changes in a user’s level of consciousness and can induce hallucinations, or visual illusions (for marijuana, this effect only occurs at high doses). Most of these drugs (except PCP) also have adrenalinelike effects on the body.

SOURCES: Schuckit, M.A. *Educating Yourself About Alcohol and Drugs: A People’s Primer.* New York: Plenum Press, 1995; and Witters, W.L., and Venturelli, P.J. *Drugs and Society.* 2d ed. Boston: Jones and Bartlett Publishers, 1988.

The problem of polydrug use raises important issues for both alcohol treatment programs and providers (Weisner 1992*b*). The use of other psychoactive drugs, such as tobacco or marijuana, may increase a person’s risk for relapse to alcohol use following alcohol treatment. Outcome studies indicate that among people in treatment for alcohol-related problems, those who use other drugs exhibit less change in their drinking habits (Brown et al. 1994; Rounsaville et al. 1987). One reason for this finding may be that polydrug use also tends to be linked to personality-behavioral types that are more resistant to change. Another important issue for clinical consideration is the possibility of drug substitution. If a person’s poly-drug use is not addressed, a reduction in alcohol use may be offset by an increase in other drug use. Furthermore, treatment for polydrug use may require different emphases or approaches than does treatment for alcohol problems alone.

That alcohol and other drug use can be considered a “dual diagnosis” reflects, in part, the fact that historically, both the treatment and research programs for these two problems developed separately and in relative isolation from each other. This separation has been reflected in treatment systems, in self-help groups (e.g., Alcoholics Anonymous versus Narcotics Anonymous and Cocaine Anonymous), and even in the scientific journals that publish clinical research findings. However, whether people initially seek treatment for alcohol or for other drug problems, they are likely to be polydrug users. Consequently, treatment must focus on both alcohol- and other drug-related problems (Brown et al. 1994). This finding presents some unique challenges for alcohol treatment providers and others interested in elements of treatment including assessment, motivation, treatment design, and outcome evaluation. This article reviews these issues by addressing polydrug use in people with alcohol-related problems.

## Assessment Issues

### Polydrug Use

Because of the high rate of comorbidity, people evaluated for alcohol-related problems also should be screened and carefully assessed for other drug use and related problems. Although additional screening does lengthen the assessment process, failure to gather drug-specific information is a serious omission. The first step in assessment is to consider each drug a person uses, whether or not its use currently meets abuse or dependence criteria. This gives a better understanding of alcohol’s interactions with other drugs in the person’s life. For example, the use of drugs other than alcohol may trigger alcohol use, and the presence of one drug in the body can dramatically alter or intensify the effect of another drug. An effective way to assess patterns and interrelationships in polydrug use is to reconstruct past AOD use on a day-by-day or days-per-month basis (Miller 1996).

### Consequences

A second step in the assessment process is to evaluate the adverse consequences (as well as the potential consequences or risks) of AOD use that have occurred during the person’s life, some of which may be quite drug-specific. Legal and health consequences, for example, vary substantially from one drug to another. Routes of drug administration also are an important consideration (e.g., risk of HIV exposure is related particularly to needle sharing). Finally, signs of intoxication and chronic-use symptoms should be assessed for various classes of drugs—for example, paranoia, hallucinations, and repetitive movements or actions (i.e., stereotypes) associated with cocaine and other stimulants.

Knowing of the mere presence or absence of any impairment, however, is less informative than understanding the specific nature and extent of AOD-use consequences in a person’s life. Risky or harm-producing use is the defining criterion for an AOD-abuse diagnosis (American Psychiatric Association [APA] 1994).

Some assessment instruments have confused consequences with other dimensions, such as dependence symptoms and help-seeking behavior. Surveying specific adverse consequences is one assessment option, although it is complicated by the person’s own perceptions of whether his or her problems are in fact the result of AOD use. A more general assessment of areas of life functioning (e.g., employment, legal, and interpersonal areas), such as that provided by the Addiction Severity Index (McLellan et al. 1990), is another alternative. (For additional approaches to assessing consequences and other dimensions mentioned here, see the review by Miller and colleagues [1995*a*], which includes methods applicable to both alcohol and other drug use.)

### Dependence

Like AOD-related consequences, AOD dependence is a continuum rather than a dichotomy. Thus, a third step in assessment is determining the *severity* of the person’s dependence on each drug used. Pharmacological issues of cross-tolerance and codependence are important clinical considerations when assessing dependence severity. Cross-tolerance is the phenomenon whereby once a person has established tolerance (i.e., relative insensitivity) for one drug, he or she also may exhibit tolerance for drugs from the same or similar classes. A person with a highly established tolerance to alcohol, for example, also may exhibit elevated tolerance to certain tranquilizers, sedatives, and anesthetics, thereby requiring larger doses of these drugs to obtain the desired effect.

The term “codependence” properly refers to simultaneous addiction (i.e., AOD dependence) to two or more drug classes. When a person stops using one drug, such as alcohol, he or she may experience a withdrawal syndrome specific to that drug. This may motivate the person to resume using the drug or to use another drug of a separate class (e.g., benzodiazepines) for which he or she has a cross-tolerance. Acute alcohol withdrawal syndrome, for example, tends to begin within 24 hours of abstinence and to run its course within approximately 1 week. Withdrawal syndrome from other drugs, however, may not begin until after 1 week or more of abstinence. Thus, a person who is dependent on multiple drugs can experience sequential or overlapping waves of withdrawal, which can interact with each other in intensifying and dangerous ways. Consequently, dependence assessment can be a critical measure in avoiding health-threatening situations.

### Functional Analysis

A fourth step in the assessment process is determining *why* the person engages in AOD use. A detailed evaluation of the antecedents, behaviors, and consequences associated with a particular behavior such as drinking (Meyers and Smith 1995), a process called functional analysis, helps to answer this question. For example, a person may be especially likely to drink excessively in the presence of certain people, when engaged in particular activities, or in specific locations, and drinking may result in rewarding consequences. When multiple drugs are involved, a separate functional analysis should be conducted for each drug, because situational factors (i.e., people, places, and activities) affecting AOD use may differ across drug classes. A person may use one kind of drug when feeling depressed or anxious, and another type when feeling bored. Discovering the functional relationships among the drugs used can make it easier to identify the person’s “primary” drug of abuse as well as provide clues on where to focus first in treatment. For example, functional analysis may reveal that a person’s alcohol use and cocaine use typically occur together, with drinking always occurring first, setting the stage for cocaine use. Alcohol may be used to enhance the effects of some drugs, such as opiates, or to “take the edge off” other drugs, such as methamphetmine. More generally, functional analysis of polydrug use can identify cues and triggers associated with AOD use, along with sources of reinforcement for maintaining the use. Such information can be particularly useful to treatment providers when planning change interventions (discussed below).

## Motivational Issues

It is important to understand a person’s motivations both for using alcohol and/or other drugs and for seeking to change such behavior. Strengthening the person’s commitment to change constitutes an important first step in treatment and can substantially improve treatment outcomes (Bien et al. 1993; Brown and Miller 1993). Motivational issues may be particularly important when screening identifies AOD users who have significant but less severe problems and dependence. Employee assistance and judicial system programs, referrals of alcohol- or drug-impaired drivers to treatment, and the widespread use of urine analysis for drug screening allow earlier identification of AOD users. People in this group usually are less motivated, however, when appearing for evaluation and treatment services.

When multiple drugs are involved, the person’s motivations for use and for change may be quite drug-specific. People often are motivated to change their use of some drugs but not others, and they may have flawed perceptions of which drugs are their “problem” or “primary” drugs. Thus, comprehensive assessment should include evaluation of a person’s motivation for change with respect to each drug used. For example, a person may be concerned and feel out of control with regard to cocaine use, somewhat aware of problems related to alcohol consumption, and unconcerned about frequent marijuana use. Such perceptions and motivations may need to be an early focus of treatment (Miller and Rollnick 1991).

Treatment goals are another important consideration, and these likewise may be quite drug specific. Because of the value of abstinence in recovery, treatment programs sometimes endorse immediate abstention from all psychoactive substances as the only viable treatment goal. Even when a person is only abusing a single drug, the likelihood of “slips” or “relapses” is high, and for people abusing multiple drugs, the risk of relapse may be even higher (e.g., Rounsaville et al. 1987). The expectation of permanently ceasing all drug use may seem at first overwhelming or impossible to achieve, and early violations may be interpreted as evidence of failure. Insistence on immediate acceptance of universal abstinence may result in poorer treatment compliance (Sanchez-Craig et al. 1984). A perspective that values progressive steps in the right direction (Marlatt et al. 1993) is consistent with research findings indicating that improvement often consists of a series of such steps interspersed with setbacks (Miller in press). Although the ultimate goal still may be cessation of all drug use, treatment may begin by targeting the person’s areas of greatest risk and concern. For example, injection drug use might be targeted first because of its high-risk potential. Consequently, a change from intravenous to oral drug use would be regarded as a positive step. This perspective is an underlying assumption in drug-substitution approaches, such as methadone maintenance. The person’s own motivation also is a crucial guide in following a step-by-step approach. At a given time, a person may be ready to stop using one drug completely but only willing to taper off the use of another. Over time, the person can establish further goals and take additional steps toward a drug-free lifestyle.

## Treatment Issues

Many similarities exist among treatments for different AOD-use disorders. Polydrug use can be treated directly through various strategies, including sobriety sampling, self-control training, and pharmacotherapy. There are also, however, unique aspects of treating alcohol problems in the context of polydrug abuse. As previously mentioned, using one drug can increase a person’s likelihood of using another drug. This phenomenon may occur simply because two drugs have been paired so often (as in the case of smoking and drinking) that the use of one drug serves as a cue for the other (for a more detailed discussion of cue responses, see the article by Shiffman, pp. 107–110). Alternatively, one drug may be used to modulate or enhance the effects of another drug. For example, alcohol may diminish judgment and inhibitions against using other drugs, such as cocaine. The abstinence violation effect of relapsing to one drug may similarly set off a chain reaction of polydrug use. Such interactions illustrate the difficulty of treating only one drug problem in the context of polydrug use. Although in the past people often were dissuaded from trying to stop smoking while recovering from alcohol problems, it may be more effective to address drug-use problems together, and failure to do so may make recovery even more difficult (Bobo et al. 1995). As discussed earlier, the proper sequencing of change and efforts that seek to decrease harm is one of the challenges of treating polydrug use and its related problems, and much remains to be learned in this area.

So how does the treatment of poly-drug abuse differ from treatment for alcohol problems alone? Certainly specific pharmacotherapies must be considered. Although drug substitution therapy, such as prescribing benzodiazepines, is almost universally rejected in treating alcohol problems, it is widely accepted as a treatment component for tobacco or opiate dependence (e.g., nicotine substitution or methadone maintenance). Naltrexone blocks the effects of opiates, and current evidence indicates that it also may suppress alcohol craving and binge drinking. In cases in which cocaine use is triggered by drinking, disulfiram may help decrease the use of both by blocking alcohol use. A variety of psychiatric medications currently are being tested as adjuncts in treating AOD abuse and dependence. Familiarity with and openness to drug-specific pharmacotherapies will be important in addressing polydrug abuse; however, this approach may create problems in alcohol treatment programs that promote a totally drug-free orientation.

Treatment setting also may play a role. For alcohol problems in general, little evidence exists to support a differential benefit from more intensive or restrictive settings, such as inpatient programs, versus outpatient treatment (Institute of Medicine 1990 and Bradley 1994). Although drug-dependence treatments have a long history of using residential therapeutic communities, it remains to be determined whether polydrug use is more effectively (and cost-effectively) treated in inpatient or residential (nonhospital) programs than with outpatient services.

Even when residential programs are used, the person ultimately is likely to return to the general community and face the challenges of coping there. In this setting, the absence of positive coping skills appears to predispose individuals to relapse, and alcohol treatment outcome research strongly supports therapies that teach effective coping strategies (Miller et al. 1995*b*). Various skills-training treatments have been tested with success. One example, the “community reinforcement approach,” is an integrated program to help people improve coping skills and establish a rewarding drug-free lifestyle (Meyers and Smith 1995). This program has been tested in both inpatient and outpatient settings and found to be effective in treating alcohol, cocaine, and heroin dependence (e.g., Higgins et al. 1993). For polydrug-use problems, it may be useful to encourage patients to acquire skills and make social-behavioral changes that will support a drug-free lifestyle rather than to focus exclusively on suppressing drug use.

Finally, there is value in having a coherent conceptual model to guide therapeutic intervention. People with polydrug-use problems typically experience a considerable amount of chaos in their lives as well as a pervasive pattern of life problems. A reactive style of counseling that focuses on week-to-week situations and crises may yield little long-term progress. Instead, the authors have found that an organized therapeutic perspective is most useful, including a long-term outlook on goals and a systematic plan for moving toward them. When offered in the context of a supportive and empathic counseling style, such a structure can impart in the patient optimism and confidence in long-term change despite short-term calamity.

## Outcome Issues

Various special issues merit consideration when evaluating the outcome of treatment for polydrug-use problems. The simplistic notion of “relapse” implies that people are always in one of two discrete and mutually exclusive states: success or failure, abstinence or unbridled use. Even when the treatment focus is a single drug, such as alcohol, this type of binary thinking is inadequate. The outcome of successful treatment is less often sudden and permanent cessation; more often, recovery is a process of longer spans of abstinence that are interrupted by ever shorter and less intense periods of use (Annis 1986; Marlatt and Gordon 1985; Miller in press). This is partially captured in the idea of a “slip,” which is by definition some period of use that does not constitute a “relapse.” Although extreme examples are clear enough, the defining characteristics of a relapse are elusive, even for a single drug.

When multiple drugs are involved, outcome evaluation becomes even more complex. Although evaluation studies commonly have examined treatment effects on one “target” drug, this approach has clear limitations. From a holistic perspective, a person may not experience a positive outcome if the suppression of alcohol consumption is offset by new or increased use of another dangerous drug, such as crack/cocaine, heroin, or tobacco. Therefore, alcohol treatment programs should be evaluated based on outcomes of other drug use as well as alcohol use. Several outcome evaluation options exist, including the previously mentioned day-by-day reconstruction of drug use. The frequency (i.e., the number of days per month) with which a person uses each drug provides a simpler index. Although quantity of use often is difficult to establish for drugs other than alcohol and tobacco, the amount of money spent on the drugs or the approximate street value of the drugs can provide at least a crude index of consumption volume. Changes in negative consequences of drug use also may prove useful as a relatively simple outcome measure (Miller et al. 1995*a*).

Finally, a binary classification of relapse and remission may not reflect the complexity of polydrug-use outcomes. Some therapeutic approaches, for example, may affect only the use of one target drug, whereas others may exert more general suppressing effects on psychoactive drug use. The longitudinal sequence of change in successful recovery also may be informative, as evidenced by current research indicating that smoking cessation is associated with more favorable alcohol treatment outcomes (Bobo et al. 1995).

## Concluding Remarks

Many of the people who seek addictions treatment experience difficulty with both alcohol and other drugs, and treatment programs are being increasingly called on to address a spectrum of drug problems. An organized professional perspective will aid those clinicians who simultaneously address polydrug-use problems to better understand the interrelationships of drinking, drug use, and the larger bio-psychosocial context in which drugs are used. The prevalence of substance abuse among those seeking medical care further warrants close integration of AOD assessment and services with primary health care (Barry and Fleming 1994).

In treating polydrug-use problems, professionals versed in state-of-the-art alcohol assessment and treatment will find much that is familiar. Although different drugs have some unique effects, risks, and consequences, there are more similarities than differences in assessment challenges and effective treatment approaches.

It seems likely that the addictions field and, ultimately, the quality of care will be strengthened by integrative efforts. It is difficult to develop sound treatment programs without a good understanding of the condition to be treated, and clearly the patterns of AOD problems change within populations over time. Rather than developing isolated methods for assessing one drug, the addictions field may benefit from understanding how alcohol, tobacco, and other drug use interact in people’s lives (Fertig and Allen 1995). Likewise, rather than merely studying the effects of treatment on the use of one drug, the field may grow by developing treatment strategies that exert a more general impact on polydrug use and related problems (e.g., Bobo et al. 1995). Such efforts may enable researchers and clinicians to step back and develop a larger perspective on the ways in which multiple drug-use resides in the psychology, biology, and sociology of human nature.

## Footnotes

1 In this article, the terms “alcohol abuse,” “alcohol dependence,” and “psychoactive drugs” refer to definitions and criteria listed in the American Psychiatric Association’s *Diagnostic and Statistical Manual of Mental Disorders, Fourth Edition.*
